# N-truncated Abeta starting with position four: early intraneuronal accumulation and rescue of toxicity using NT4X-167, a novel monoclonal antibody

**DOI:** 10.1186/2051-5960-1-56

**Published:** 2013-09-06

**Authors:** Gregory Antonios, Nasrin Saiepour, Yvonne Bouter, Bernhard C Richard, Anders Paetau, Auli Verkkoniemi-Ahola, Lars Lannfelt, Martin Ingelsson, Gabor G Kovacs, Thierry Pillot, Oliver Wirths, Thomas A Bayer

**Affiliations:** 1 Georg-August-University Goettingen, University Medicine Goettingen, Division of Molecular Psychiatry, 37075 Goettingen, Germany; 2 U4 Graduate School, The Aging Brain, Goettingen, Germany; 3 Department of Pathology, University and University Hospital of Helsinki, Helsinki, Finland; 4 Department of Neurology, Helsinki University Central Hospital, Helsinki, Finland; 5 Department of Public Health/Geriatrics, Uppsala University, Uppsala, Sweden; 6 Institute of Neurology, Medical University of Vienna, AKH4JA-1097 Vienna, Austria; 7 SynAging, 54000 Nancy, France

**Keywords:** Pyroglutamate Abeta, Abeta oligomer, Toxicity, Arctic, Swedish, Presenilin-1, 5XFAD, Transgenic mouse model, Familial Alzheimer’s disease, Sporadic Alzheimer’s disease, Abeta 4-40, Abeta 4–42

## Abstract

**Background:**

The amyloid hypothesis in Alzheimer disease (AD) considers amyloid β peptide (Aβ) deposition causative in triggering down-stream events like neurofibrillary tangles, cell loss, vascular damage and memory decline. In the past years N-truncated Aβ peptides especially N-truncated pyroglutamate Aβ_pE3-42_ have been extensively studied. Together with full-length Aβ_1–42_ and Aβ_1–40_, N-truncated Aβ_pE3-42_ and Aβ_4–42_ are major variants in AD brain. Although Aβ_4–42_ has been known for a much longer time, there is a lack of studies addressing the question whether Aβ_pE3-42_ or Aβ_4–42_ may precede the other in Alzheimer’s disease pathology.

**Results:**

Using different Aβ antibodies specific for the different N-termini of N-truncated Aβ, we discovered that Aβ_4-x_ preceded Aβ_pE3-x_ intraneuronal accumulation in a transgenic mouse model for AD prior to plaque formation. The novel Aβ_4-x_ immunoreactive antibody NT4X-167 detected high molecular weight aggregates derived from N-truncated Aβ species. While NT4X-167 significantly rescued Aβ_4–42_ toxicity *in vitro* no beneficial effect was observed against Aβ_1–42_ or Aβ_pE3-42_ toxicity. Phenylalanine at position four of Aβ was imperative for antibody binding, because its replacement with alanine or proline completely prevented binding. Although amyloid plaques were observed using NT4X-167 in 5XFAD transgenic mice, it barely reacted with plaques in the brain of sporadic AD patients and familial cases with the Arctic, Swedish and the presenilin-1 *PS1Δ9* mutation. A consistent staining was observed in blood vessels in all AD cases with cerebral amyloid angiopathy. There was no cross-reactivity with other aggregates typical for other common neurodegenerative diseases showing that NT4X-167 staining is specific for AD.

**Conclusions:**

Aβ_4-x_ precedes Aβ_pE3-x_ in the well accepted 5XFAD AD mouse model underlining the significance of N-truncated species in AD pathology. NT4X-167 therefore is the first antibody reacting with Aβ_4-x_ and represents a novel tool in Alzheimer research.

## Background

Several hypotheses have been proposed and competed in trying to explain the underlying cause of Alzheimer’s disease (AD). The dominant hypothesis, since 1991, is the amyloid hypothesis that implicates amyloid-β (Aβ) deposits as the cause of this common neurodegenerative disorder. Extracellular deposits of Aβ protein and the intracellular accumulation of phosphorylated tau protein are the basis of the neuropathological characterization of AD [1-3]. Contemporary AD research has been driven forward through the use of advanced molecular biology tools. One key discovery, the isolation and sequencing of the gene encoding the larger amyloid precursor protein (APP) [4], was made possible by the biochemical analysis of β-amyloid containing blood vessels (CAA, cerebral amyloid angiopathy) [5] and amyloid plaques consisting of Aβ [6].

The “Amyloid Hypothesis” was proposed, therefore, based on the previously mentioned discovery [3,7]. Since then, however, amyloid plaque load in the brain and cognitive impairment in suffering patients [8] or even in transgenic mouse models for AD [9,10] have not been found to be consistently correlated. This gave rise to considerable controversy in the field.

Memory loss in AD is the most prominent clinical manifestation of the disease. To that end, Haass and Selkoe [11] have recently evaluated the concept that soluble oligomers of Aβ, acting as diffusible assemblies, are capable of interfering with synaptic function and integrity. This has provided a gateway for understanding the basis of memory loss in AD. They debated that while insoluble plaque deposits might function as reservoirs of the pathological oligomers, the small soluble oligomers affect synaptic structure and plasticity. A modified amyloid hypothesis was brought forth, wherein it has been suggested that intraneuronal Aβ accumulation precedes the extracellular formation of Aβ plaques and other AD pathological events [12]. It is now well accepted that the pathologically inert amyloid fibrils, which are found in plaques, originate from a nearly irreversible reaction driven by monomeric Aβ peptide through toxic protofibrillar intermediates. For instance, the fact that amyloid plaques possibly are major sources of soluble toxic Aβ-aggregates that could readily be activated by exposure to biological lipids, has been credibly demonstrated by Martins et al. [13].

In addition to Aβ_1_ starting with aspartate as the first amino acid, several N-truncated and modified Aβ species have been characterized [14-16]. In fact, various N- and C-terminal variants have been described in conjunction with *in vitro* and *in vivo* analysis of amyloid deposits in AD [14,17,18]. The toxicity of Aβ was further promoted due to enhanced aggregation and deposition brought on by the increase in C-terminal length of Aβ (from Aβ_x-40_ to Aβ_x-42_) and by N-terminal truncation [19-21]. Among Aβ species present in AD plaques, Lewis et al. [22] reported that Aβ_4-42_ is a relatively abundant species in AD, aged controls and vascular dementia patients.

Mori and colleagues discovered that approximately 15-20% of Aβ peptides carried a pyroglutamate residue at their N-terminus [23]. This ignited a spark of interest in the temporal and spatial deposition of pyroglutamate Aβ, which has increased ever since. For instance, Saido et al. demonstrated by immunohistochemistry and biochemical assays Aβ_pE3-x_ is present in equivalent or larger amounts than full-length Aβ in senile plaques. The suggestion that Aβ_pE3-x_ precedes the deposition of unmodified Aβ (Aβ_1-x_) was also proposed by the authors based on their analysis of brain tissue from Down syndrome cases [24]. Saido et al. furthermore suggested that, due to their limited degradation, Aβ_pE3_ and other modified Aβ species accumulate unhindered [16]. The aggregation tendency and stability of the Aβ_pE3-x_ peptides is due to the formation of the lactam ring and loss of two negative charges and one positive charge [16]. The stability of the peptide is further increased by the formation of the moiety-terminal pyroglutamate that is resistant to degradation by peptidases. He and Barrow [19] reported that, as compared to full-length Aβ, Aβ_pE3-x_ peptides exhibited enhanced β-sheet formation and aggregation propensity in aqueous and hydrophobic media. They proposed that a reduction of the level of unfavorable charge repulsion between strands, brought on by the loss of the three charged groups, facilitates and stabilizes β-sheet formation. Using immunoprecipitation in combination with mass spectrometry, Portelius and colleagues [25] showed that Aβ_1-40_, Aβ_1-42_, pyroglutamate Aβ_pE3-42_ and Aβ_4-42_ can be detected in the hippocampus and cortex of AD patients. Interestingly, it has been demonstrated that N-terminal deletions enhance Aβ aggregation when comparing Aβ_4-42_ with Aβ_1-42_[21].

The weak correlation between the severity of dementia and the density and localization of amyloid plaques in the brain of AD patients is one of the major flaws in the amyloid hypothesis. Even before the primary signs of plaque deposition, memory impairment and pathological changes already appear in many AD mouse models [26]. Soluble oligomers are low molecular weight non-fibrillar structures, which are stable in aqueous solution and remain soluble even after high speed centrifugation [26]. Occurring more often than their proliferation inside the extracellular space, Aβ oligomers develop preferentially within neuronal processes and synapses [27,28]. Results from several labs led to the proposition of these oligomers as the missing link in the amyloid hypothesis. While Aβ plaques are poor correlates for the clinical symptomatology in AD and Down syndrome patients, soluble oligomers are suggested to be good predictors for synaptic loss [29], neurofibrillary tangles [30] and clinical phenotype [31,32]. Tomiyama et al. generated APP transgenic mice expressing the E693Δ mutation, which causes neuronal cell death and cognitive impairment by enhanced intracellular Aβ oligomerization without plaque formation [33].

Although Aβ_4–42_ is highly abundant in AD brains and was the first N-truncated peptide discovered [14] its possible role in AD pathology has been largely overlooked. We have recently shown that Aβ_4–42_ rapidly forms aggregates and possesses a high aggregation propensity [34]. *In vitro* and *in vivo* exposure indicated that Aβ_4-42_ is as toxic as Aβ_pE3-42_ and Aβ_1–42_. In addition, we have generated transgenic mice expressing Aβ_4-42_ (Tg4-42 transgenic line) that developed a massive CA1 pyramidal neuron loss in the hippocampus [34]. Interestingly, as assessed using the Morris water maze test, the hippocampus-specific expression of Aβ_4–42_ alone correlated with age-dependent spatial reference memory deficits [34]. In the present report, we developed a novel antibody specific for N-truncated Aβ and characterized it using a toxicity assay, 5XFAD transgenic mice, sporadic and familial AD cases.

## Methods

### Generation of NT4X-167 antibody

The novel oligomeric Aβ specific antibody NT4X-167 (IgG2b; official name of cell line Aβ_4–40_ NT4X-167; DSM ACC3162) was generated by immunizing three Balb/c mice with unconjugated Aβ_4–40_. After preparation of the lymph nodes they were fused with the myeloma cell line P3-X63-Ag8 for generation of the hybridoma cells. The hybridoma supernatants of mixed clones were screened by ELISA and immunohistochemistry and subcloned. The idea behind the generation of novel oligomeric antibodies was that in solution Aβ_4–40_ peptides are forming stable aggregates that can be used as an epitope for antibodies that specifically bind at the N-terminus of Aβ_4–40_. Therefore Aβ_4–40_ was used for immunizing mice and positive clones screened in four steps. After fusion, the hybridoma cells were screened by an enzyme-linked immune-absorbent assay (ELISA) for antibody production that (1) bind Aβ_4–10_ and (2) Aβ_4–40_, but (3) not Aβ_36–40_. Positive antibody clones were further screened by immunohistochemical staining of human brain sections. (4) The last step of the screening procedure was that they should not preferentially bind to amyloid plaques thereby identifying NT4X-167.

### Electrophoresis and blotting of synthetic peptides

For Western blot analysis under reducing conditions, peptides were loaded on 4-12% Tris-Tricin VarioGels (Anamed), transferred to 0.45 μm nitrocellulose membranes (GE Healthcare) and detected using the primary antibodies IC16 (1 μg/ml), 1–57 (1 μg/ml) and NT4X-167 (1 μg/ml). Blots were developed using Luminata Crescendo Western HRP Substrate (Millipore) and exposed with the ODYSSEY Fc (LI-COR).

For Western blotting under native conditions 4-16% SERVAGel N native gels (Serva) were used under blue native conditions. Running and transfer buffers were applied according to the manufacturer instructions. Nitrocellulose membranes (GE Healthcare) were detected using the primary antibodies IC16 (1 mg/ml; diluted 1:1000) (generous gift by Sascha Weggen [35]), 1–57 (1 mg/ml; diluted 1:500) [36] and NT4X-167 (1 mg/ml; diluted 1:300). Secondary antibodies were rabbit-anti-mouse HRP-conjugated (Dianova). Blots were developed using Luminata Crescendo Western HRP Substrate (Millipore) and exposed with the ODYSSEY Fc (LI-COR).

### Monomerization of synthetic peptides

Stock solutions of synthetic peptides (1mg/ml in 10 mM NaOH; PSL, Heidelberg) were prepared, sonicated for 5 min in water bath (Sonorex RK 100H, Bandelin electronic), quickly frozen in liquid nitrogen and stored at −80°C.

### Pepscan of synthetic peptides using ELISA

100 ng 16 amino acid long Aβ peptides (Aβ_1–16_, _2–17_, _3–18_, _4–19_, _5–20_, _6–21_, _7–22_, _8–23_, _9–24_ and _10–25_) were used. The peptides were coated in a 96 well plate overnight and reacted with NT4X-167 as primary antibody followed by incubation with horse radish peroxidase conjugated secondary antibodies (Dianova).

### Neuronal culture

Cortical neurons from embryonic day 16–17 Wistar rat fetuses were prepared as previously described [37]. In brief, dissociated cortical cells were plated at 50,000 cells/well in 48-well plates precoated with 1.5 mg/mL polyornithine (Sigma). Cells were cultured in a chemically defined Dulbecco’s Modified Eagle’s/F12 medium free of serum (Gibco) and supplemented with hormones, proteins and salts. Cultures were kept at 35°C in a humidified 5% CO2 atmosphere, and at 6–7 DIV, cortical population was determined to be at least 97% neurons by immunostaining as done previously [38]. At 6 DIV, the medium was removed and cortical neurons were incubated for 24 h with vehicle (cell culture medium) or Aβ peptides (dissolved in cell culture medium) at the indicated concentrations.

### Cell viability measurement

Following a 24 h incubation of primary cortical neurons with Aβ peptides, cell viability was determined using a calcein-AM assay (Invitrogen, Molecular Probes). Briefly, cells were washed twice with PBS and incubated protected from light for 30 min at room temperature in the presence of 2 μM calcein-AM solution prepared in PBS. Cells were then washed twice with PBS and incubated for 15 min at room temperature in PBS containing 1% Triton X-100 (v/v). The level of calcein fluorescence was monitored by fluorescence emission at 530 nm after exciting at 485 nm, using a Fluostar microplate reader (BMG-Labtechnologies, France).

### Transgenic mouse brain samples

5XFAD mice express the 695 amino acids isoform of the human amyloid precursor protein (APP695) carrying the Swedish/London/Florida mutations under the control of the murine Thy1-promoter [39]. In addition, human presenilin-1 (PS1) carrying the M146L/L286V mutations is expressed also under the control of the murine Thy1-promoter. 5XFAD mice used in the current study were backcrossed for more than eight generations to C57Bl/6J wild-type mice to obtain an incipient congenic line on a C57Bl/6J genetic background [40]. Homozygous 5XFAD mice were verified by back-crossing to wildtype mice. All animals were of male sex and handled according to guidelines of the German animal protection law.

### Human brain samples

Human brain samples were obtained from the Netherlands Brain Bank (NBB), the Institute of Neurology, Medical University of Vienna, Austria, Department of Pathology, University of Helsinki, Finland and Department of Pathology, University of Uppsala, Sweden were approved by the local Ethical Committees.

### Immunohistochemistry of brain sections

Human and mouse tissue samples were processed as described previously [36]. In brief, 4 μm paraffin sections were pretreated with 0.3% H_2_O_2_ in PBS to block endogenous peroxidases and antigen retrieval was achieved by boiling sections in 0.01 M citrate buffer pH 6.0, followed by 3 min incubation in 88% formic acid. Primary antibodies were incubated overnight, followed by incubation with biotinylated secondary rabbit-anti-mouse antibodies (DAKO) before staining was visualized using the ABC method with Vectastain kit (Vector Laboratories) and diaminobenzidine as chromogen. Primary antibodies used were IC16 (against the N-terminus of Aβ_1-x_ 1 mg/ml; diluted 1:5000), 1–57 (against the N-terminus of pyroglutamated Aβ_3-x_, 1 mg/ml; 1:5000) and NT4X-167 (against the N-terminus of Aβ_4-x_; 2 mg/ml; diluted 1:200).

### Statistical analysis

Differences between groups were tested with one-way analysis of variance (ANOVA) followed by Bonferroni multiple comparison. All data are given as means ± standard error of the mean (SEM). All statistics were calculated using GraphPad Prism version 5.04 for Windows (GraphPad Software, San Diego, California, USA) and SPSS statistics version 17.0 (IBM, Armonk, New York, USA).

## Results

### Specificity of NT4X-167 binding for Aβ under denaturing conditions

**Figure 1:**
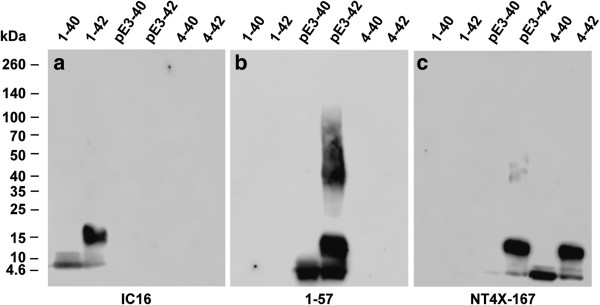
**SDS-PAGE Western blot analysis of IC16, 1–57 and NT4X-167 antibodies. Freshly dissolved synthetic Aβ variants (7 μg each) were probed to a membrane. (a)** IC16 detects Aβ_1–40_ and Aβ_1–42_ monomers and low molecular weight aggregates. **(b)** 1–57 recognizes Aβ_pE3-40_ and Aβ_pE3-42_ monomers, low molecular weight and larger aggregates of Aβ_pE3-42_. **(c)** NT4X-167 recognizes monomers and low molecular weight aggregates derived from Aβ_pE3-40_, Aβ_pE3-42_, Aβ_4–40_ and Aβ_4–42_.

Freshly dissolved Aβ peptides were subjected to SDS-PAGE to dissect the binding specificity of the three tested antibodies (Figure 1). Under denaturing conditions NT4X-167 reacted with both N-terminally truncated Aβ_pE3-X_ and Aβ_4-X_ variants, but not with Aβ_1-X_. In addition to monomers and dimers, trimers and tetramers of Aβ_pE3-42_ and Aβ_4–42_ were recognized. Aβ_pE3-40_ and Aβ_4–40_ produced primarily monomers and dimers. Antibody 1–57 stained only Aβ_pE3-40_ and Aβ_pE3-42,_ but no other bands as previously demonstrated [36]. IC16 recognized the N-terminus of full-length Aβ_1–40_ and Aβ_1–42_, but not any of the N-truncated peptides.

### Specificity of NT4X-167 binding for Aβ under native conditions

**Figure 2:**
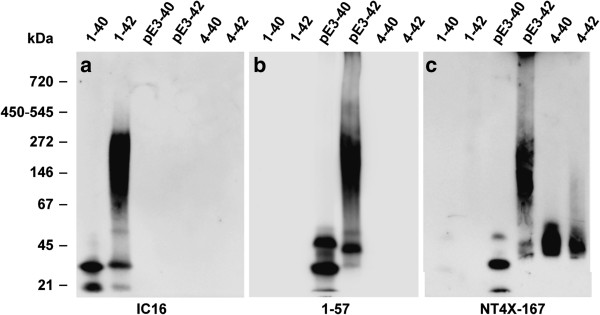
**Western blot analysis of IC16, 1–57 and NT4X-167 antibodies under native conditions.** Freshly dissolved synthetic Aβ variants (2 μg each) were probed to a membrane. **(a)** IC16 detects Aβ_1–40_ and Aβ_1–42_ monomers, low molecular weight oligomers and larger aggregates of Aβ_1–42_. **(b)** 1–57 recognizes Aβ_pE3-40_ and Aβ_pE3-42_ monomers, low molecular weight oligomers and larger aggregates of Aβ_pE3-42_. **(c)** NT4X-167 recognizes monomers and aggregates derived from Aβ_pE3-40_, Aβ_pE3-42_, Aβ_4–40_ and Aβ_4–42_.

Under native conditions (Figure 2), freshly dissolved Aβ peptides immediately formed high molecular weight aggregates of different sizes. The binding specificity of the three antibodies tested was the same as in the SDS PAGE. IC16 was specific for the N-terminus of full-length Aβ, but did not bind with any of the other N-truncated peptides. 1–57 detected only Aβ_pE3-40_ and Aβ_pE3-42_. NT4X-167 reacted with all four N-truncated Aβ peptides Aβ_pE3-40_, Aβ_pE3-42_, Aβ_4–40 and_ Aβ_4–42_. The different Aβ variants produced distinct bands corresponding to approximately: Aβ_1–40_ (20 and 30 kDa), Aβ_1–42_ (20, 30 and 55 k Da as well as larger aggregates >70 kDa), Aβ_pE3-40_ (20, 30 and 50 kDa), Aβ_pE3-42_ (30, 50 and 55 kDa as well as larger aggregates >70 kDa). Interestingly, Aβ_4–40 and_ Aβ_4–42_ elicited only one band at approximately 50 kDa. The distinct band sizes are only an approximation based on the migration of the protein ladder under native conditions.

### Epitope mapping using pepscan ELISA

**Figure 3:**
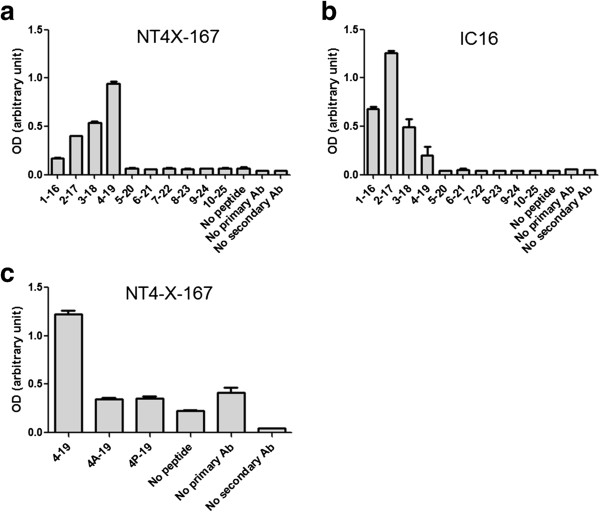
**Phenylalanine at position four of Aβ is important for NT4X-167 binding.** Pepscan analysis using ELISA of **(a**, **c)** NT4X-167 and **(b)** IC16. Soluble synthetic Aβ peptides of 16 amino acids in length were probed and incubated with the antibodies. **(a)** NT4X-167 recognizes N-terminal truncated Aβ peptides corresponding to positions 2–4 of Aβ. Peptides starting with phenylalanine (P; position 4 of Aβ) gives the highest signal. No signal above background is seen in the absence of probed peptide and primary or secondary antibody. **(b)** IC16 binds to peptides with N-terminal Aβ_1–4_ with a preference for position Aβ_2_. **(c)** NT4X-167 signal is completely abolished by replacing phenylalanine with alanine (A) or proline (P) showing the importance of phenylalanine at position 4 for NT4X-167 binding. The signals of mutant peptides are at background signals. Abbreviation: Ab, antibody.

Pepscan assays (Figure 3) were performed in order to identify the binding epitope of NT4X-167 and IC16 to the primary Aβ peptide sequences. The N-terminal binding specificity of 1–57 has already been published [36]. Pepscan ELISA for signal detection revealed that the binding site of NT4X-167 ranged between N-truncated Aβ_2–4_ with the highest signal for N-truncated Aβ_4-x_ starting with phenylalanine at position four, as compared to IC16, which preferentially bound to positions 1–3 of Aβ. Mutational analysis of Aβ_4–19_ replacing phenylalanine with alanine (Aβ_4A-19_) or proline (Aβ_4P-19_) completely inhibited binding of NT4X-167 antibody. Therefore, phenylalanine at position four of Aβ is the essential amino acid required for NT4X-167 antibody binding.

### NT4X-167 detects Aβ_4–42_ in the low picomolar range

In order to analyze the sensitivity of NT4X-167, a dilution series was performed with freshly dissolved synthetic Aβ_4–42_ and the staining was visualized using a Western blot under reducing conditions (Additional file 1: Figure S1). NT4X-167 detected monomers and dimers between 1 and 0.03 μg corresponding to a minimum of approximately 7 picomoles of Aβ_4–42_.

### NT4X-167 rescued Aβ_4–42_*in vitro*

**Figure 4:**
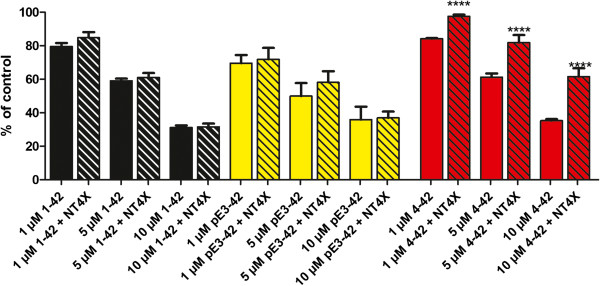
**Cellular toxicity of N-truncated Aβ**_**x-42 **_**peptides and treatment effect of NT4X-167. In rat primary cortical neurons, all Aβ**_**x-42 **_**peptides induce significant dose-dependent cellular toxicity.** NT4X-167 treatment completely rescues toxic effects of freshly dissolved 1, 5 and 10 μM Aβ_4–42_, but not of Aβ_pE3-42_ or Aβ_1–42_*in vitro* (ANOVA, P<0.0001, F= 87.24, dF=17). After ANOVA, the individual groups were subsequently analyzed using Bonferroni multiple comparisons. Abbreviation: ****, P<0.0001.

*In vitro* toxicity was studied in primary neurons using a calcein assay. Treating the cells with freshly prepared Aβ_4-42_, Aβ_pE3-42_ and Aβ_1-42_ resulted in a dose-dependent reduction in cell viability (Figure 4) as previously shown [34]. While NT4X-167 significantly rescued toxicity of Aβ_4-42_, no effect was observed after Aβ_pE3-42_ or Aβ_1-42_ exposure. The *in vitro* toxicity assay provided compelling evidence that NT4X-167 specifically protected against Aβ_4-42_ and not with Aβ_pE3-42_ aggregates.

### NT4X-167 detected early intraneuronal Aβ accumulation in 5XFAD transgenic mice

**Figure 5:**
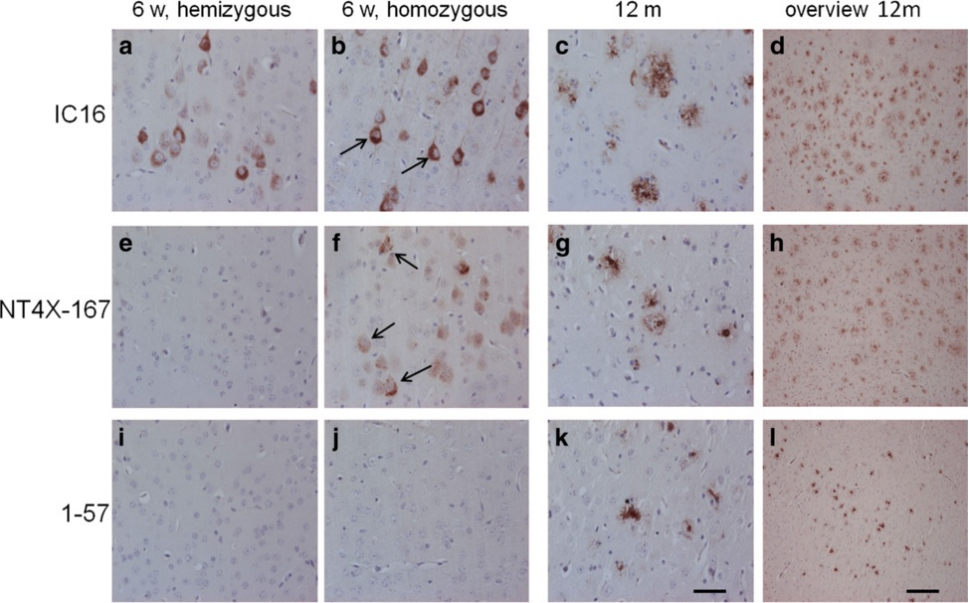
**Immunohistochemical staining of cortical sections of 5XFAD transgenic mice. (a)** Immunostaining with IC16 demonstrating intraneuronal Aβ accumulation in 6 week old hemizygous 5XFAD. **(b)** Homozygous 5XFAD mice exhibited more intensive intraneuronal staining at the same age. **(c**, **d)** Abundant extracellular plaque staining with IC16 at the age of 12 months. **(e)** No signal was detected using NT4X-167 in 6 week old hemizygous 5XFAD. **(f)** In homozygous 5XFAD mice significant intraneuronal staining was observed with NT4X-167. **(g**, **h)** Abundant extracellular plaque staining with NT4X-167 at the age of 12 months. **(i**, **j)** No intraneuronal Aβ was observed with the pyroglutamate specific antibody 1–57 in 6 week-old hemizygous **(i)** and homozygous **(j)** 5XFAD mice. **(k**, **l)** Extracellular plaque staining with 1–57 at the age of 12 months. At 12 months only hemizygous 5XFAD are used. Abbreviations: w, week; m, month. Scale bar in **k** for **a**-**c**, **e**-**g** and **i**-**k**: 50 μm, and in **l** for **d**, **h** and **l**: 200 μm.

In order to compare the staining pattern of NT4X-167 (against Aβ_4-x_ and pyroglutamate Aβ_3-x_), 1–57 (against Aβ_pE3-x_), and IC16 (against Aβ_1-x_), hemizygous and homozygous 5XFAD mice were studied using cortical sections, as this is the brain area with known abundant intraneuronal Aβ in this model [39,40]. Homozygous 5XFAD mice were generated in order to observe an aggravated amyloid pathology at an earlier time point as compared to hemizygous mice. As expected, intraneuronal Aβ_1-x_ accumulation was observed in young (Figure 5a and b), but not in aged 5XFAD mice (Figure 5c and d). Such transient appearance of intraneuronal accumulation of Aβ peptides in young APP transgenic has already been described earlier [41]. Homozygous 6 week-old 5XFAD mice showed an aggravated intraneuronal signal compared to hemizygous mice (Figure 5a vs. 5b). Aged mice demonstrated abundant Aβ_1-x_ accumulation in amyloid plaques (Figure 5c and d). NT4X-167 recognized intraneuronal Aβ in 6 week-old homozygous 5XFAD mice (Figure 5f), a signal absent with the Aβ_pE3-x_ specific antibody 1–57 (Figure 5j). Therefore the signal was due to Aβ_4-x_ accumulation, which represents the earliest N-truncated Aβ species. In fact, using 1–57, we did not see any intraneuronal signal at any ages analyzed. Amyloid plaques were detected with both NT4X-167 and 1–57 in aged mice (Figure 5g-h, k-l).

### NT4X-167 demonstrated a minor plaque binding activity in sporadic and familial Alzheimer’s disease

**Figure 6:**
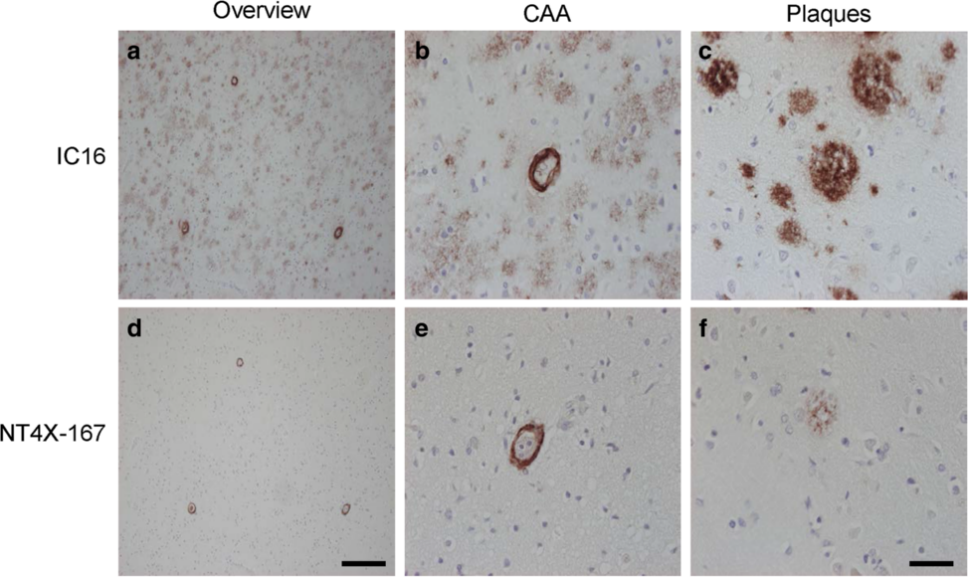
**Immunohistochemical staining pattern in superior temporal gyrus of a sporadic AD brain. (a**-**c)** IC16 antibody visualized cerebral amyloid angiopathy (CAA) and plaques. **(d**-**f)** Staining of parallel sections shows that NT4X-167 recognized preferentially CAA rather than plaques. Scale bar: **a**, **d**: 200 μm and **b**, **c**, **e**, **f**: 50 μm.

**Figure 7:**
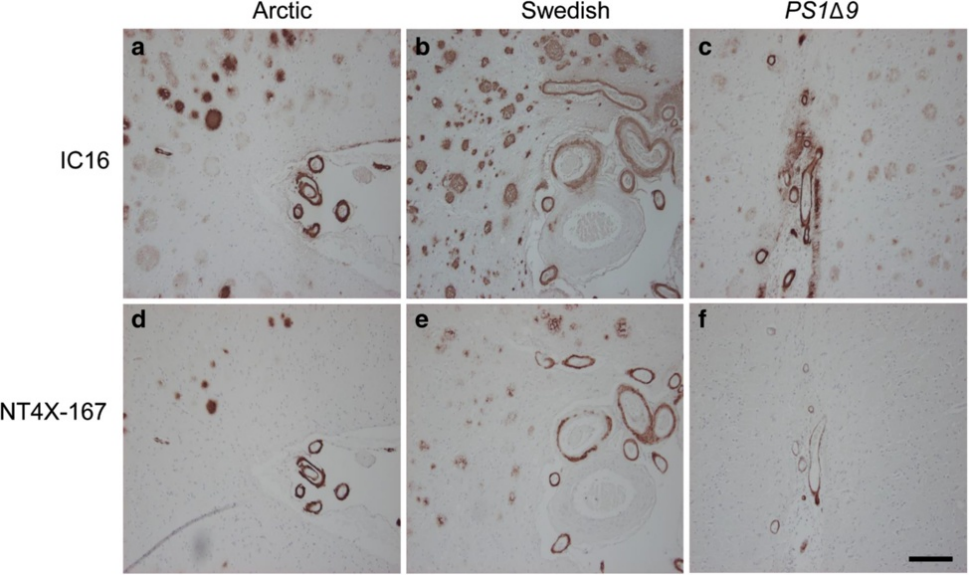
**Immunohistochemical staining of cerebral cortex in patients with familial AD. (a**-**c)** using IC16 antibody and **(d**-**f)** parallel sections using NT4X-167 antibody. **(a**, **d)** The patient harboring the Arctic mutation elicited positive blood vessels (CAA) and plaques with both antibodies, staining in plaques being less pronounced with NT4X-167. **(b**, **e)** The patient with the Swedish mutation also demonstrated positive blood vessels and plaques with both antibodies and again with a weaker staining in plaques with NT4X-167. **(c**, **f)** In the patient with the PS1 mutation ∆Exon9, positive blood vessels and plaques were seen with both antibodies again with a less pronounced staining in plaques with NT4X-167. Scale bar: 200 μm.

In order to characterize the staining pattern of the NT4X-167 in AD patients, cortical tissue sections with sporadic (Table 1, Figure 6) and familial AD (Table 2, Figure 7) were analyzed. Compared to the IC16, NT4X-167 recognized only a minor portion of plaques in brain tissue of AD patients. Cerebral amyloid angiopathy (CAA) staining of blood vessel walls was seen with both antibodies. In familial AD cases, NT4X-167 positive plaques were almost absent in patients with a mutation in presenilin-1 gene (*PS1Δ9*; [42]), and much weaker in cases with the Arctic [43,44] or Swedish [45] APP mutation compared to IC16 staining.

**Table 1:** List of demographic data of sporadic AD patients and non-demented controls and the staining profile of the antibodies

	**No**	**Age Mean±SEM**	**Sex M/F**	**Braak stage**	**ApoE4**	**Plaques (IC16)**	**CAA (IC16)**	**Plaques (NT4X-167)**	**CAA (NT4X-167)**
Sporadic AD	13	76 ± 3	3/10	4-6	7/13	13/13	13/13	3/13	13/13
Controls	10	80 ± 2	6/4	0-1	2/10	5/10	3/10	0/10	3/10

Of note, none of the controls showed NT4X-167 staining of plaques although 5 of them were positive with IC16, demonstrating a clear difference between AD and control cases. The amount of NT4X-167-positive plaques in sporadic AD brain was low.

*Abbreviations: No* number of cases, *M* male, *F* female, *ApoE4* number of cases with at least one ApoE4 allele, *CAA* cerebral amyloid angiopathy.

**Table 2:** List of the demographic data and staining profile of the antibodies in familial AD patients

**Gene**	**Mutation**	**Sex**	**Age**	**Plaques (IC16)**	**CAA (IC16)**	**Plaques (NT4X-167)**	**CAA (NT4X-167)**
*APP*	Arctic	M	64	+	+	+	+
	Swedish	F	61	+	+	+	+
*PS1*	*PS1*Δ*9*	M	61	+	+	+	+
		M	64	+	+	+	+
		M	69	+	+	+	+

NT4X-167 showed only CAA but almost no plaques in *PS1*Δ*9* patients, while both CAA and plaques were observable in patients with Arctic and Swedish mutations in the APP gene.

*Abbreviations: M* male, *F* female, *CAA* cerebral amyloid angiopathy.

### NT4X-167 did not cross-react with other major proteinopathies

**Figure 8:**
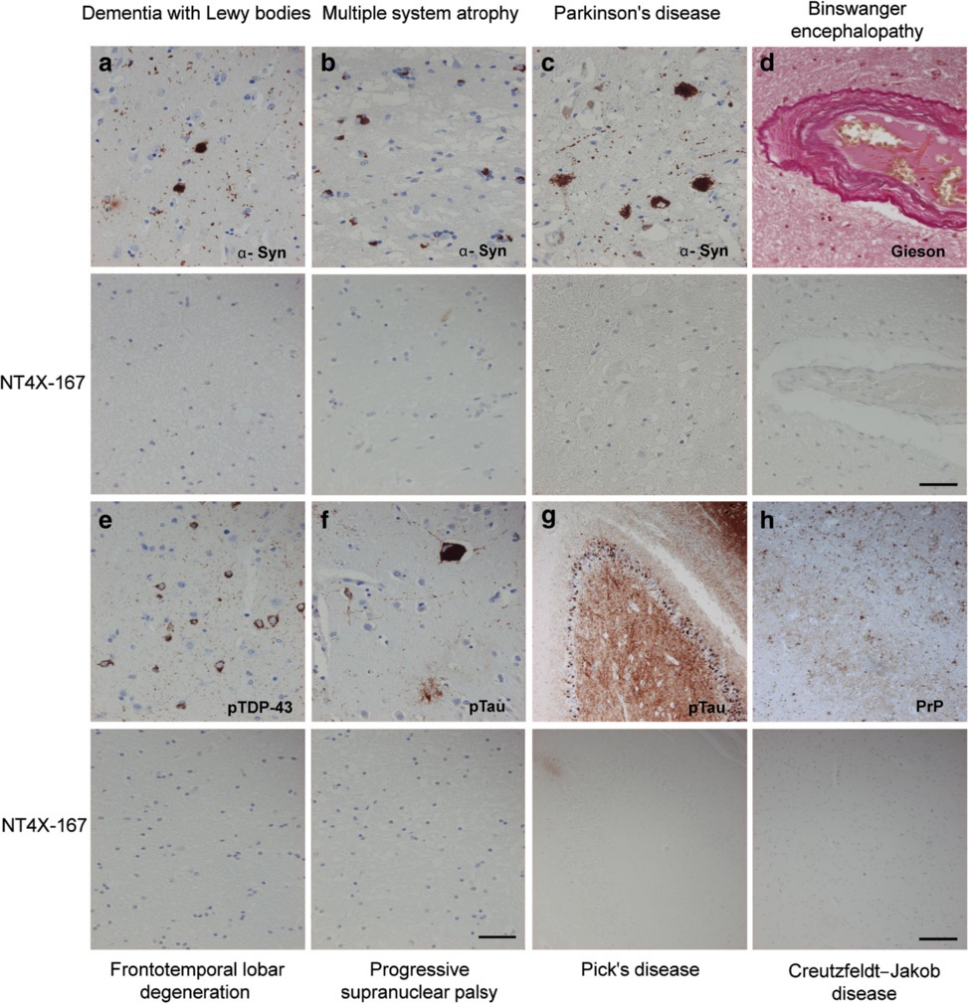
**NTX4-167 showed no cross-reactivity with diagnostic aggregates in other major neurodegenerative diseases.** Lower panels represent parallel sections stained with NT4X-167. **(a**-**c)** Alpha-synuclein (α-syn) positive aggregates in Lewy bodies and Lewy neurites in dementia with Lewy bodies and Parkinson disease and glial inclusions in multiple system atrophy. **(d)** Pathological vessels in Binswanger encephalopathy (subcortical arteriolosclerotic encephalopathy). **(e)** Phospho-TDP-43 immunoreactive neuronal cytoplasmic inclusions in a patient with frontotemporal lobar degeneration. **(f**-**g)** Phospho-tau (pTau) immunoreactive aggregates in progressive supranuclear palsy and in Pick’s disease. **(h)** Prion protein (PrP) immunopositive synaptic deposits in sporadic Creutzfeldt-Jakob disease. Scale bar in **a**-**f** 50 μm, **g**-**h**, 200 μm.

In order to study a potential cross-reactivity with other disease-typical aggregates, brain tissue sections were stained with disease-specific markers and compared with NT4X-167 reactivity. NT4X-167 did not cross-react with other aggregated deposits of non-AD neurodegenerative disorders. The following pathological structures showed no immunoreactivity (Table 3; Figure 8): (i) Phospho-Tau immunoreactive structures, including tufted astrocytes in progressive supranuclear palsy (PSP) and Pick bodies in Pick’s disease (PiD). (ii) α-Synuclein immunopositive Lewy bodies (brainstem and cortex) and Lewy neurites in Parkinson’s disease (PD) and dementia with Lewy bodies (DLB), and glial cytoplasmic inclusions in multiple system atrophy (MSA). (iii) Phospho-TDP-43 immunoreactive neuronal cytoplasmic and neuritic deposits in frontotemporal lobar degeneration with TDP-43 pathology (FTLD-TDP), amyothrophic lateral sclerosis (ALS), and AD with limbic TDP-43 deposits. (iv) Prion protein (PrP) immunopositive amyloid plaques, synaptic, plaque-like, and perineuronal deposits. In addition, there was no immunoreactivity associated with small vessel disease in Binswanger disease.

**Table 3:** Demographic data and examined anatomical regions from other neurodegenerative disorders cases

**No**	**Case/Disease**	**Age**	**Sex**	**Examined regions**
1	DLB	81	F	Temporal Cx
2	PD	62	M	Mesencephalon (SN)
3	MSA	52	M	Pons
4	PSP	69	M	Basal Ganglia
5	PiD	70	F	Hippocampus + Ent Ctx + Temp Ctx
6	FTLD	62	F	Hippocampus + Ent Ctx + Temp Ctx
7	CJD	72	F	Hippocampus + Ent Ctx + Temp Ctx + Cbll
8	Binswanger disease	49	F	Basal Ganglia

*Abbreviations: DLB* dementia with Lewy bodies, *PD* Parkinson’s disease, *MSA* multiple system atrophy, *PSP* progressive supranuclear palsy, *PiD* Pick’s disease, *FTLD* frontotemporal lobar degeneration, *CJD* Creutzfeldt-Jakob disease, *M* male, *F* female, *Ent Cx* entorhinal cortex, *Temp Ctx* temporal cortex, *SN* substantia nigra, *Cbll* cerebellum.

## Discussion

The amyloid-β hypothesis has been the most influential hypothesis in coining the molecular pathology of AD [2]. According to the initial hypothesis, amyloid fibrils, which are large insoluble polymers of Aβ found in senile plaques, are the major trigger of neuron loss and dementia that are typical for AD. While Aβ plaques are poor correlates for the clinical symptomatology in AD and Down syndrome patients, soluble oligomers are suggested to be good predictors for synaptic loss [29], neurofibrillary tangles [30] and clinical phenotype [46]. Furthermore, memory impairment and pathological changes in many AD mouse models occur well before the onset of plaque deposition [47]. Albeit there are convincing genetic, biochemical and cell biological data pointing to a major role of Aβ in AD, growing evidence points towards soluble Aβ oligomers rather than Aβ precipitated in plaques. Blennow et al. [48] for example discussed whether Aβ deposition is the cause or consequence of neurodegeneration in sporadic AD, but also whether the transgenic mouse models are at all accurate models for sporadic AD.

Soluble oligomers are low molecular weight non-fibrillar structures, which are stable in aqueous solution and remain soluble even after high speed centrifugation. Aβ oligomers develop preferentially within neuronal processes and synapses rather than in the extracellular space [27,28]. At high concentrations, vesicular full-length Aβ aggregates form high molecular weight oligomers which are capable of seeding amyloid fibril growth [49]. Results from several labs propose these oligomers to be the missing link in the amyloid hypothesis. Just like in the human brain, studies using AD mouse models support the pathogenic role of oligomers. In the Tg2576 mouse model, the appearance of Aβ dodecamers coincided with the onset of spatial memory impairment. Interestingly, injection of these purified oligomers into the ventricle of wildtype rats caused a dramatic drop in spatial memory performance [50]. With regard to short-term effects, oligomers have been shown to impair synaptic plasticity by blocking long term potentiation and reinforcing long term depression [51]. Another hint was reported by Tomiyama et al. [33], who generated APP transgenic mice expressing the E693Δ mutation causing neuronal cell death and cognitive impairment by enhanced intracellular Aβ oligomerization without plaque formation. Loss of Aβ clearance instead of increased Aβ generation has been considered to be involved in the pathology of the sporadic variant of AD [52]. The above mentioned thoughts consider full-length Aβ_1–40/1–42_ as the major culprit in AD pathology.

Of note, full-length Aβ peptides are physiological molecules produced throughout the life of a human being. The generation of N-truncated Aβ peptides has been suggested to increase toxicity [53]. Pike et al. [21] compared Aβ peptides with initial residues at positions 1, 4, 8, 12, and 17 and ending with residue 40 or 42 and showed that N-terminal deletions enhance Aβ aggregation in relation to full-length Aβ. Furthermore they reported that Aβ peptides exhibiting aggregation showed circular dichroism spectra consistent with predominant β-sheet conformation, fibrillar morphology under transmission electron microscopy, and significant toxicity in cultures of rat hippocampal neurons.

We have recently extended these observations and showed that soluble aggregates have specific features responsible for their neurotoxicity [34]. Aβ_4-40_, Aβ_4-42_, Aβ_1-42_ and Aβ_pE3-42_ were unstructured in the monomeric state [34]. However, upon heating the Aβ variants showed a high propensity to form folded structures, in particular the three most toxic variants Aβ_pE3-42_, Aβ_1-42_ and Aβ_4-42_. In addition, monomeric Aβ_4-42_ and Aβ_pE3-42_ were rapidly converted to soluble aggregated species. Both N-truncated variants exhibited similar biochemical properties, which opens the discussion which one of them might be more important in AD pathology [34].

In the present report we endeavored to address this question. We succeeded to develop an antibody differentiating between full-length Aβ and the two other major N-truncated variants, Aβ_4-x_ and Aβ_pE3-x_. In combination with two other antibodies exclusively reacting with Aβ_1-x_ (IC16) or Aβ_pE3-x_ (1–57), we were able to show that Aβ_4-x_ preceded Aβ_pE3-x_ accumulation in the brain of 5XFAD transgenic mice. More importantly, Aβ_4-x_ was detected together with Aβ_1-x_ in the intraneuronal compartment of cortical neurons prone to degenerate in 5XFAD mice at 12 months of age [39,40]. Early and transient intraneuronal accumulation of Aβ correlated with subsequent neuron loss also in diverse APP/Aβ transgenic mouse models and brain regions [34,41,54-57]. Interestingly, such a transient appearance of intraneuronal Aβ_x-42_ has also been described by Mori et al. [58] studying the brain of Down syndrome patients between 3 to 73 years. Using an antibody against the N-terminus of Aβ_pE3-x_, no intraneuronal staining was reported [58] corroborating our observation of a lack of intraneuronal accumulation of Aβ_pE3-x_ in 6 week-old 5XFAD mice in the present study.

Using an *in vitro* toxicity assay, we were able to demonstrate that NT4X-167 is particularly protecting against Aβ_4–42_ and that the binding to Aβ_pE3-42_ has no therapeutic consequence. The mechanism(s) of the diverging biological effects are not clear. The Western blot analysis might not accurately reflect the difference in affinity of NT4X-167 between Aβ_4–42_ and Aβ_pE3-42_. The data from the *in vitro* toxicity assay provides evidence that NT4X-167 preferentially binds Aβ_4–42_. On the other side it could also be that NT4X-167 does not efficiently bind to some toxic aggregate(s) of Aβ_pE3-42_ as it did not significantly detect the aggregate at 50 kDa as compared to 1–57 antibody under native conditions. We have previously shown that passive immunization of 5XFAD mice with 9D5, a monoclonal antibody specifically detecting low molecular weight Aβ_pE3-x_ aggregates, significantly reduced overall Aβ plaque load and Aβ_pE3-x_ levels, and normalized behavioral deficits [59].

While amyloid plaques were observed using NT4X-167 in 5XFAD transgenic mice, it barely reacted with plaques in the brain of sporadic AD patients and familial cases with the Arctic, Swedish and the presenilin-1 mutation *PS1∆9*. These data are corroborated by a previous work by Kuo et al. [60]. They analyzed Aβ pathology using chemical and morphological approaches comparing the plaques of APP23 transgenic mice and human AD brain. The authors concluded that despite an apparent overall structural resemblance to AD pathology, the chemical analyses revealed that the amyloid plaque cores in APP23 transgenic mice were completely soluble in buffers containing SDS [60]. Human AD plaque cores were highly resistant to chemical and physical disruption accounting for the extreme stability of AD plaque cores [60]. Moreover, the corresponding lack of post-translational modifications such as N-terminal degradation, isomerization, racemization, pyroglutamyl formation, oxidation, and covalently linked dimers in transgenic mouse Aβ, provides an explanation for the differences in solubility between human AD and the APP23 mouse plaques [60]. NT4X-167 preferably stained Aβ in blood vessels in human specimens, in which Aβ_x-40_ is a major component. The Aβ plaques in PS1Δ9 AD cases are characterized by cotton wool morphology composed by Aβ_x-42_ aggregates. The lack of cotton wool plaque staining using NT4X-167 further strengthens the possibility that it may prefer binding to Aβ_4-40_ as compared to Aβ_4-42_ aggregates.

Selkoe and others reported that toxic Aβ oligomers are primarily dimers and trimers of Aβ [28,61,62]. Haass and Selkoe argued that small molecules that can specifically inhibit the formation of Aβ oligomers and/or prevent their binding to and stabilization on neuronal membranes is at the top in the search for an AD therapy [11]. More recently, De Strooper [63] discussed that it is more likely that several of the identified oligomeric species (derived from full-length Aβ) have similar or overlapping properties. They conclude that coexistence of several oligomeric populations that do or do not propagate into fibrils is possible. Despite the differences in structure, stability and concentration, all oligomers may contribute to Aβ toxicity. They further discussed some technical issues defining oligomers like the apparent ‘SDS resistance’ [63]. Bitan et al. [64] have demonstrated that SDS can artificially induce oligomerization of Aβ. Hepler et al. [65] were able to isolate monomers, trimers and tetramers as major bands derived from full-length Aβ oligomers, Aβ fibrils and Aβ monomers after SDS-PAGE separation. Our data are well in line with these previous observations. Under reducing conditions Aβ_pE3-40_ and Aβ_4–40_ generated monomers and dimers, while Aβ_pE3-42_ and Aβ_4–42_ in addition produced trimers and tetramers as previously shown [14,59]. Using native conditions, Aβ_1–42_ and Aβ_pE3-42_ appeared as aggregates of different sizes with higher molecular weight aggregates. In contrast Aβ_4–40_ and Aβ_4–42_ ran as a single band at approx. 50 kDa.

In fact, analysis of amyloid deposits in AD brains revealed various N- and C-terminal variants [14,17,18]. The increased C-terminal length of Aβ (from Aβ_x-40_ to Aβ_x-42_) enhances its aggregation properties. Faster aggregation leads to earlier Aβ deposition, which is believed to promote its toxicity [20,21,66]. Recently, Aβ_1-43_ was discovered as a novel toxic peptide in AD [67,68]. Besides Aβ peptides starting with aspartate as the first amino acid (Aβ_1_), several N-truncated and modified Aβ species have also been described [14-16,69]. Aβ_4–42_ being one of them is particularly interesting as its discovery dates back to 1985 by Masters et al. [14]. Lewis et al. [22] reported that Aβ_4-42_ is a relatively abundant species in AD, aged controls and vascular dementia patients. Using immunoprecipitation in combination with mass spectrometry, Portelius and colleagues [25] showed that Aβ_1-40_, Aβ_1-42_, Aβ_pE3-42_ and Aβ_4-42_ can be detected in the hippocampus and cortex of AD patients. Moreover, it has been demonstrated that N-terminal deletions enhance Aβ aggregation comparing Aβ_4-42_ with Aβ_1-42_[21]. Youssef et al. [38] showed that Aβ_1-42_ and Aβ_pE3-42_ exhibited similar effects on neuronal cytotoxicity in primary cortical neurons and on memory impairment after intracerebroventricular injection in wildtype mice. Aβ_pE3-42_ is now an established factor contributing to AD pathology [53] and may even be aggravating the severity of the disease [70]. Sergeant et al. demonstrated that amino-truncated Aβ species represented more than 60% of all Aβ species, not only in full blown AD, but also, and more interestingly, at the earliest stage of AD pathology [71]. They concluded, that a vaccine specifically targeting these pathological amino-truncated species of Aβ_x-42_ are likely to be promising, by inducing the production of specific antibodies against pathological Aβ products that are, in addition, involved in the early and basic mechanisms of amyloidosis in the human brain.

The importance of position four of Aβ is corroborated by Haupt et al. [72], who observed an N-terminal β-strand, previously assumed to be an unstructured region [73-77]. Using proline mutagenesis to probe the structural relevance of N-terminal residues, they demonstrated that mutations affecting residues 4 or 8, significantly increased the fraction of elongated aggregates indicating that disrupting the N-terminal β-strand favors protofibrils relative to oligomers [72]. The pathological impact of Aβ_4–42_ is elucidated by the generation of transgenic mice (Tg4-42) expressing Aβ_4–42_[34]. The Tg4-42 mice develop a severe age-dependent spatial reference memory deficit and massive hippocampus neuron loss.

At present, the enzymes responsible for N-terminal truncation are not well studied. Aminopeptidase A contributes to the N-terminal truncation of Aβ peptide producing Aβ_2-x_[78]. Saido et al. [16] suggested that mono- or dipeptidylaminopeptidases cleave Aβ_1-x_ producing N-terminal truncated Aβ_3-x._ Aβ_pE3-x_ formation is catalyzed by glutaminyl cyclase [79-82]. Which enzymes are involved in the truncation steps to generate Aβ_3-x_ and Aβ_4-x_ is unknown.

## Conclusion

The present report describes the binding properties of the novel antibody NT4X-167, which recognizes the N-terminus of N-truncated Aβ. NT4X-167 bound most efficiently to Aβ_4-x_. Phenylalanine at position four of Aβ was imperative for NT4X-167 binding. *In vitro* toxicity experiments demonstrated that Aβ_4–42_ induced neuron death was significantly rescued by NT4X-167 treatment. No rescue effect was observed for Aβ_1–42_ or Aβ_pE3-42_ toxicity. NT4X-167 detected only a minor fraction of plaques in brain from sporadic and familial AD patients and 5XFAD transgenic mice. It preferentially reacted with intraneuronal Aβ in young 5XFAD mice. The finding that Aβ_4-x_ precedes Aβ_pE3-x_ in the well accepted 5XFAD AD mouse model further underlines the significance of Aβ_4-x_. NT4X-167 did not cross-react with aggregates typical for other major neurodegenerative disorders implicating that the recognized aggregates are specific for AD. Taking all observations together, NT4X-167 represents a novel tool for AD research and therapy.

## Competing interests

A patent application for NT4X-167 was filed by the University Medicine of Goettingen and TAB.

## Authors’ contributions

TAB is the PI of this study, conceived and designed the experiments and contributed to the interpretation of findings and writing of manuscript. GA and NS performed experiments and drafted the manuscript along with TAB. YB, BCR, TP, and OW performed experiments and contributed to revising the manuscript. AP, AVA, LL, MI, GK collected samples and characterized human disease samples used in the current study. All authors read and approved the final manuscript.

## Supplementary Material

Additional file 1: Figure S1 SDS-PAGE Western blot analysis of Aβ_4-42_ for sensitivity testing of NT4X-167 using freshly dissolved peptides. NT4X-167 detects monomers and dimers of Aβ_4-42_ up to 0.03 μg peptide. Click here for file
