# Supplementary material for: N-truncated Abeta starting with position four: early intraneuronal accumulation and rescue of toxicity using NT4X-167, a novel monoclonal antibody
Source: Acta Neuropathol Commun. 2013 Sep 6;1:56. doi: 10.1186/2051-5960-1-56 (PMC3893517; doi:10.1186/2051-5960-1-56)

**Additional file 1: Figure S1.** SDS-PAGE Western blot analysis of Aβ_4-42_ for sensitivity testing of NT4X-167 using freshly dissolved peptides. NT4X-167 detects monomers and dimers of Aβ_4-42_ up to 0.03 µg peptide.


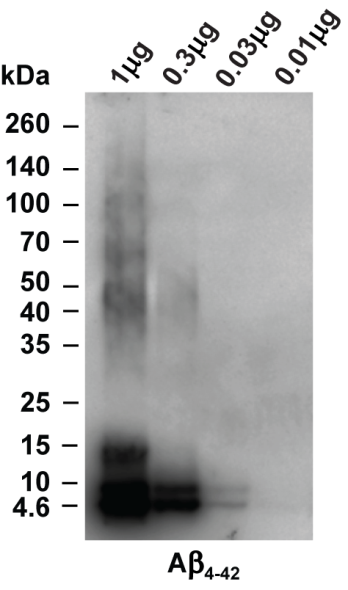

Supplement: Additional file 1: Figure S1 — SDS-PAGE Western blot analysis of Aβ4-42 for sensitivity testing of NT4X-167 using freshly dissolved peptides. NT4X-167 detects monomers and dimers of Aβ4-42 up to 0.03 μg peptide. [file 2051-5960-1-56-S1.docx]
